# Practical application and evaluation of an integrated training pathway for mental health literacy and clinical communication skills for undergraduate dental students based on simulation-based training

**DOI:** 10.3389/fmed.2025.1712035

**Published:** 2026-01-06

**Authors:** Yao Wang, Lanlan Ye, Meiqin Zhou, Xi Chen

**Affiliations:** Medical School, Lihu Campus, Shenzhen University, Shenzhen, Guangdong Province, China

**Keywords:** dental education, mental health literacy, clinical communication skills, simulation-based teaching, VR training, standardized patient (SP)

## Abstract

**Objective:**

To construct and evaluate a comprehensive training pathway based on simulated operation training to improve the mental health literacy and clinical communication skills of junior dental undergraduate students.

**Methods:**

A quasi-randomized controlled pre-post mixed-methods design was used, with 60 lower-year dental students enrolled. The intervention group received 5-module training (VR scenario cognition, stress regulation, simulation integration, standardized patient communication, reflective reinforcement), while the control group received conventional teaching. Assessments were conducted at T0 (baseline), T1 (post-Module 3), T2 (post-Module 5), and T3 (1 month post-internship) using tools including DANVA-2, MHL-Q, SEGUE, JSE-HP, and CD-RISC-10.

**Results:**

At T3, the intervention group showed significantly higher scores than the control group: DANVA-2 accuracy (80.7% ± 6.1% vs. 66.2% ± 6.5%, Cohen’s *d* = 2.31), MHL-Q (71.1 ± 4.8 vs. 60.8 ± 5.4, *d* = 2.05), SEGUE (80.9 ± 5.9 vs. 67.2 ± 6.0, *d* = 2.28), and CD-RISC-10 (28.4 ± 3.5 vs. 23.2 ± 3.7, *d* = 1.46) (all *p* < 0.05). Emotional recognition (OR = 1.12, 95%CI: 1.06–1.18) and communication effectiveness (OR = 1.15, 95%CI: 1.09–1.21) independently predicted clinical integration ability.

**Conclusion:**

The simulation-based training pathway improves dental students’ MHL, CC skills, and psychological resilience, and correlates with better clinical performance, providing empirical support for dental education.

## 1 Introduction

Clinical communication skills and mental health literacy (MHL) are core competency requirements in global medical education, and their level directly affects the quality of doctor-patient relationships and the safety of diagnosis and treatment (1). The invasive nature and high anxiety-inducing characteristics of dental treatment often lead patients to experience psychological reactions such as fear and depression. However, dental students’ insufficient psychological resilience and communication skills may not only increase the resistance to treatment but also raise the risk of medical disputes. Therefore, integrating MHL with clinical communication skills into undergraduate dental education has become an important direction in medical education reform (2–4). Dental clinical practice involves intricate procedures, extended patient contact time, and high patient anxiety levels. Particularly during dental treatments, patients often exhibit nervousness, fear, or even treatment refusal (5). Surveys indicate that approximately 50% of dental patients experience varying degrees of treatment anxiety, which may not only hinder smooth medical procedures but also diminish treatment outcomes and increase the risk of doctor-patient conflicts. Additionally, the confined working space in oral procedures demands high levels of concentration and precise technical skills from practitioners. Prolonged meticulous operations can easily lead to physician fatigue and stress (6). Despite the growing recognition of the importance of mental health for medical students, domestic medical schools still lack sufficient emphasis on mental health education (7). Current undergraduate dental education in China prioritizes technical skill development while neglecting students’ MHL and communication competencies, lacking systematic, context-based integrated teaching approaches (8). Students struggle to manage patients’ complex emotions and unexpected situations during clinical practice, straining the doctor-patient relationship and potentially triggering medical disputes. Prolonged exposure to high-pressure work environments also predisposes dental students to psychological issues (9). Traditional communication courses predominantly rely on theoretical lectures, disconnecting from practical clinical skill training. Students often lack effective coping strategies when confronting real patients’ complex emotions and unexpected situations (10). Therefore, there is an urgent need for a teaching model that integrates clinical skills with MHL, enabling repeated training in safe, controlled environments. This approach is essential to meet the demands for cultivating holistic, comprehensive professional competence in future dental practitioners.

Internationally, the medical education community has long emphasized cultivating medical students’ MHL and communication skills (11). Research by Sekhar et al. (10) also supports enhancing medical students’ MHL, empathy, communication, and adaptability through school-based mental health programs. This study evaluated a school-based mental health program during psychiatric rotations, aiming to integrate theoretical knowledge with practical skills through experiential learning in real community settings (10). In dental education, He et al. (8) explored the feasibility of integrating patient-doctor communication education into Taiwan’s dental curriculum, emphasizing the necessity of improving communication to optimize treatment outcomes and patient satisfaction.

There has long been a structural issue in China’s dental education characterized by an emphasis on technical skills over professional (literacy) (10, 11). Courses on MHL and clinical communication are mostly scattered across humanities elective courses, lacking contextualized integrated practice. During internships, students often face the dual predicament of patient resistance and low self-efficacy due to insufficient ability to recognize emotions and a lack of diverse communication strategies (12, 13). Existing interventions mostly focus on improving single skills and have not yet formed a complete training system covering the “cognition-practice-reflection” process (13, 14).

This study integrates MHL and clinical communication skills training throughout the entire simulation-based training process. Experiential learning enhances the efficiency of skill transformation through a cyclic mechanism of “concrete experience—reflective observation—conceptual construction—active practice.” Its value in medical education has been confirmed, and it is particularly suitable for literacy cultivation in simulation training scenarios (15, 16). However, there are still significant gaps in existing research: first, internationally, MHL and communication skills training are mostly carried out independently, and there is a lack of dental-specific evidence for their integration mechanisms and synergistic effects; second, domestic studies are mostly cross-sectional surveys, lacking intervention verification with quasi-randomized controlled designs, and failing to clarify the long-term stability of training effects; third, the implementation paths of simulation training are mostly limited to the single module of standardized patients (SP), and integrated multi-dimensional programs combining VR scenarios, stress regulation, etc., have not yet been reported. Based on this, this study constructs a training path consisting of 5 modules including VR scenario cognition, stress regulation, and simulation integration. Through a quasi-randomized controlled trial, it systematically evaluates its impact on dental undergraduates’ MHL, clinical communication skills, and psychological resilience, aiming to provide a replicable practical paradigm for literacy cultivation in dental education.

## 2 Methods

### 2.1 Research design

This study employed a quasi-randomized controlled, pre-post mixed-methods design (Quantitative + Qualitative Mixed Methods). Quantitative assessments were conducted at four time points: baseline (T0), completion of Module 3 (T1), completion of Module 5 (T2), and 1 month post-clinical rotation (T3). Qualitative interviews supplemented the quantitative analysis. The study process involved the intervention group receiving the simulation-based integrated training pathway, while the control group received conventional instruction. Both groups completed identical measurements and evaluations at the same time points.

### 2.2 Ethical approval and informed consent

This study strictly adheres to the Declaration of Helsinki, has been approved by the Medical Ethics Committee of Shenzhen University (Approval Number: SZUMC-2024-035), and has passed the review of the University’s Science and Education Department and the Medical College. Before the start of the study, all participants were informed of the research purpose, procedures, risks, and benefits through both oral and written means, and they signed informed consent forms; participants were aware that their participation was voluntary, that they could withdraw at any time, and that this would not affect their course grades.

### 2.3 Study population and sample size

#### 2.3.1 Grouping method

The study population comprised first- and second-year undergraduate dental students at a medical college. Quasi-randomization grouping with classes as units: After sorting the 6 classes by student ID, a statistician independent of the study generated a random sequence using the Excel random number table. Classes with odd-numbered sequences were included in the intervention group (3 classes, 30 cases), and classes with even-numbered sequences were included in the control group (3 classes, 30 cases). After grouping, blinding of group information was implemented for researchers and evaluators (only the statistician knew the grouping results), and the balance between groups was verified through comparison of baseline data (Table 1) to ensure no selection bias.

**Table 1:** Baseline data of two groups of undergraduate dental students.

Indicator	Intervention group (*n* = 30)	Control group (*n* = 30)	*p*
Gender (Male/Female)	16/14	15/15	0.80
Age (years)	20.1 ± 0.8	20.3 ± 0.9	0.37
Academic performance (GPA)	3.42 ± 0.31	3.39 ± 0.29	0.70
DANVA-2 accuracy (%)	68.5 ± 12.3	67.8 ± 11.9	0.82
MHL-Q total score	52.7 ± 6.8	53.2 ± 7.1	0.65
SEGUE score	72.3 ± 8.5	71.8 ± 9.1	0.83
JSE-HP score	102.4 ± 11.2	101.9 ± 10.8	0.86
PSS-10	19.8 ± 4.1	20.1 ± 3.9	0.77
CD-RISC-10	25.3 ± 5.7	24.9 ± 5.3	0.78
OSCE baseline score	76.5 ± 9.2	75.8 ± 8.7	0.76

The verification of the balance of baseline data between the two groups eliminates confounding factors for the subsequent analysis of intervention effects, ensuring that the differences between the groups can be attributed to the different training paths.

Inclusion criteria were: (1) Voluntarily participate and sign the informed consent form; (2) Pass the mental health screening (using the Symptom Checklist-90 (SCL-90), with a total score <160 points and each factor score <2 points, and confirm no history of mental illness based on past medical records); (3) Have no severe physical diseases (such as limb dysfunction) and be able to complete all training and assessments; (4) Have an attendance rate of ≥80% during the study period. Exclusion criteria were: (1) Failure to pass the SCL-90 screening or a history of mental illnesses such as depression and anxiety; (2) Attendance rate <80% during the study period or failure to complete all assessments; (3) Withdrawal from the study midway due to personal reasons.

#### 2.3.2 Sample size estimation

A power analysis was conducted using G*Power 3.1 software with the following assumptions: (1) Significance level *α* = 0.05 (two-tailed test); (2) Test power (1 − *β*) = 0.8; (3) Referring to previous similar intervention studies in dental education (8), a large effect size (Cohen’s *d*) of 0.8 was anticipated for the primary outcome measure (total score of MHL-Q). Based on our pilot data with a standard deviation of 5.0, the expected mean difference between groups was 4.0 points. (4) The expected difference in total MHL-Q scores between groups was 8 points, with a standard deviation of 5.0. The calculation showed that at least 25 cases were needed in each group. Considering a 20% attrition rate, a total of 60 cases were finally included.

### 2.4 Training pathways and instructional units

The intervention group underwent a simulation-based training pathway integrating MHL and clinical communication skills, structured into five modules. The first module focused on VR scenario recognition, utilizing a virtual reality case library to simulate varying degrees of patient anxiety. Students were required to identify and label emotional states. The second module addressed stress regulation, teaching mindful breathing and brief pre-procedure warm-up rituals, with feedback training via heart rate monitoring devices. Module 3 integrated simulation practice by embedding anxiety scenarios into Simodont dental simulations. A voice system randomly triggered events like sudden patient mouth closure to train students’ coping and reassurance techniques. Module 4 featured Standardized Patient (SP) communication training, simulating patients refusing treatment or exhibiting emotional fluctuations within Objective Structured Clinical Examination settings, requiring students to achieve effective communication within time constraints. Module 5: Reflection and Reinforcement. Students document emotional triggers, communication strategies, and causes of procedural errors, then engage in peer-supervised reflection and improvement. The control group followed traditional teaching methods, including theoretical lectures and routine skill training, without incorporating emotion recognition, stress regulation, or SP simulation components (Table 2). Both groups were taught by the same batch of teachers: All teachers received unified training (including interpretation of the intervention plan, standardization of traditional teaching content, and specifications for the use of assessment tools). The duration and frequency of teaching were consistent (twice a week, 90 min each time, for a total of 8 weeks) to avoid bias caused by differences in teacher qualifications.

**Table 2:** Training pathways and teaching units.

Module	Content	Integrated design	Tools/Methods
U1	VR contextual cognition	Identify patient’s micro-expressions of anxiety → Label emotion types	VR dental case library (with anxiety behavior parameters)
U2	Stress regulation	Mindful breathing + “1-min pre-surgery” warm-up ritual	Heart rate monitoring wristband feedback
U3	Integrated simulated operation	Responding to the anxious event of “patient suddenly closing mouth” during Simodont filling	Voice command system triggers unexpected scenarios
U4	SP communication training	Handling anxious patients who refuse treatment in OSCE	Standardized patient (SP) scripts
U5	Reinforced reflection	Analyzing emotional triggers for operational errors through emotion diaries	Structured diary template + Peer supervision

### 2.5 Evaluation indicators and measurement tools

This study was conducted in Chinese. Assessments were carried out at four time points: baseline (T0), after completing Module 3 (T1), after completing Module 5 (T2), and 1 month into clinical clerkship (T3). Quantitative indicators encompassed emotional recognition ability, MHL, communication effectiveness, empathy capacity, stress perception, stress resilience, and clinical performance. All standardized measurement tools used have been validated and adapted for the Chinese population: the DANVA-2 facial expression recognition test (validated for Chinese young adults), MHL-Q (adjusted according to Chinese dental education context with pre-survey verification), SEGUE framework scoring (adapted for Chinese clinical communication scenarios), Chinese version of JSE-HP (reliability and validity verified in domestic medical student groups), PSS-10 and CD-RISC-10 (widely used in Chinese psychological research with mature psychometric properties), OSCE (Objective Structured Clinical Examination) scoring (consistent with national medical education assessment standards in China), and mentor-based clerkship evaluations (customized based on domestic clinical training requirements) (Table 3).

**Table 3:** Evaluation indicators and measurement tools.

Dimension	Indicator	Tool
MHL	Emotion recognition ability	DANVA-2 facial expression recognition test (Baseline/T2)
	Knowledge-attitude-practice	MHL-Q (T0–T3)
CC	Communication effectiveness	SEGUE framework score (T1–T3)
	Empathy ability	JSE-HP
Psychological resilience	Perceived stress	PSS-10 (T0/T2)
	Stress recovery ability	CD-RISC-10 (T0/T2)
Clinical performance	Integrated ability	OSCE scenario score (Operation + Communication ratio 6:4)
	Practical performance	Preceptor rating (Operation fluency + Patient soothing effectiveness)

### 2.6 Data collection and management

**Figure 1:**
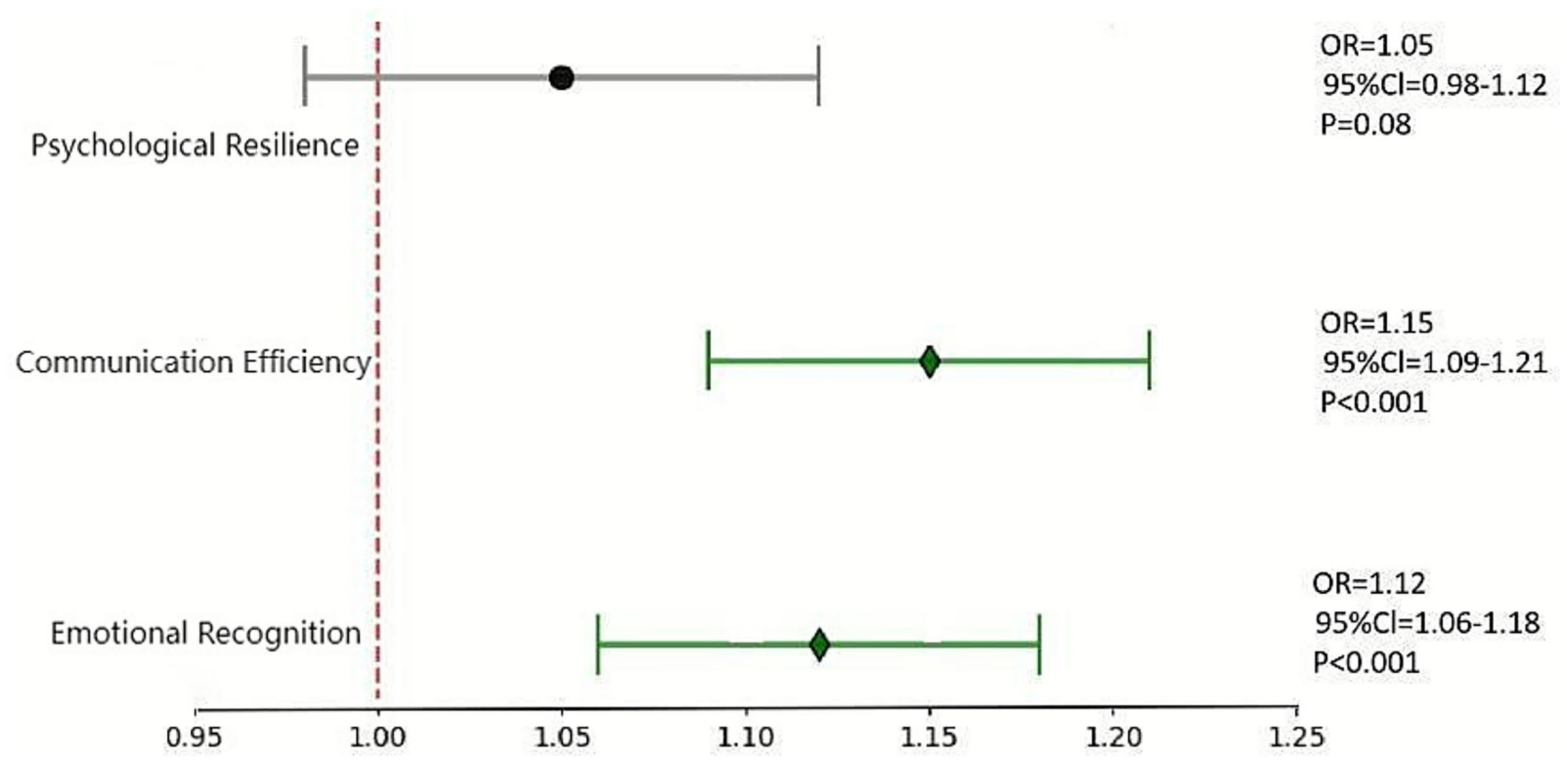
Multivariate logistic regression analysis chart of influencing factors of clinical integration ability Axis labels: X-axis = Predictors (emotional recognition ability, communication effectiveness, psychological resilience); Y-axis = Odds ratio (OR) and 95% confidence interval (error bars); Horizontal line = OR = 1 (no predictive effect). Interpretation: The OR values of emotional recognition ability and communication effectiveness are both >1, and their 95% CIs do not cross the horizontal line, with *p* < 0.001, indicating that for each 1-unit increase in either of them, the probability of meeting the standard for clinical integration ability increases by 12 and 15% respectively; the OR value of psychological resilience is close to 1, and its 95% CI crosses the horizontal line, showing no significant predictive effect.

A PRISMA-compliant flow diagram (Figure 1) is included to illustrate the full process of recruitment, screening, grouping, and multi-timepoint assessments (T0-T3) for both the intervention and control groups.

#### 2.6.1 Quantitative data collection

Questionnaires were distributed and collected on-site by research assistants who received standardized training (effective recovery rate = 100%). OSCE scores were independently evaluated by 2 dental clinical teachers, with the average score used as the final result. Preceptor ratings (including operation fluency and patient soothing effectiveness) were assessed by 3 senior clinicians using the “Clinical Internship Scoring Form,” with a Kappa coefficient of 0.82 indicating good inter-rater reliability.

#### 2.6.2 Qualitative data collection

Students in the intervention group submitted 1 structured reflective journal per week throughout the training. After the clinical internship, semi-structured interviews were conducted in 3 rounds (10 students per round). All interview recordings were transcribed verbatim and anonymized (names replaced with S1-S30 to protect privacy).

#### 2.6.3 Data management

Quantitative data were double-entered into the SPSS 26.0 database to ensure accuracy. Qualitative data (reflective journals and interview transcripts) were imported into Nvivo 12.0 software for thematic coding and analysis.

### 2.7 Statistical analysis

#### 2.7.1 Quantitative analysis

Measurement data conforming to a normal distribution are expressed as “mean ± standard deviation (x ± s).” Independent sample *t*-tests are used for comparisons between groups, and paired *t*-tests are used for comparisons before and after within groups; Count data are expressed as “cases (%),” and comparisons are made using the *χ*^2^ test. Multivariate logistic regression analysis was used to identify factors influencing clinical integration ability, and ROC curves were plotted to evaluate predictive efficacy, with a test level of *α* = 0.05. Qualitative analysis: The thematic analysis method by (26) was adopted. Two researchers independently coded the data (Cohen’s kappa = 0.86). Core themes were extracted through the process of “coding—refining themes—verifying consistency,” and triangulation was conducted in combination with quantitative data.

## 3 Results

### 3.1 Baseline comparability

A total of 60 junior undergraduates majoring in stomatology were included, with 30 cases in the intervention group and 30 cases in the control group. There were no statistically significant differences between the two groups in terms of gender, age, academic performance (GPA), and all baseline assessment indicators (emotion recognition ability, MHL-Q, SEGUE, JSE-HP, PSS-10, CD-RISC-10, and baseline OSCE scores) (all *p* > 0.05). The balance between the groups was good, and they were comparable (Table 1).

### 3.2 Mental health literacy-related indicators

#### 3.2.1 Emotion recognition ability (DANVA-2)

The intervention group showed a significant improvement in emotion recognition accuracy after completing Module 3 (T1) compared to baseline (T0) (78.3% ± 6.2% vs. 65.4% ± 5.8%, *p* < 0.001). This enhanced level was maintained at T2 and T3 (T2: 82.1% ± 5.9%, T3: 80.7 ± 6.1%). The control group showed no significant changes at corresponding time points (T0: 64.9% ± 6.0%, T3: 66.2 ± 6.5%, *p* > 0.05). Comparisons between groups revealed the intervention group significantly outperformed the control group at T2 and T3 (*p* < 0.001) (Table 4).

**Table 4:** Changes in mental health literacy indicators (mean ± SD).

Time point	Intervention group DANVA-2 (%)	Control group DANVA-2 (%)	Intervention group MHL-Q	Control group MHL-Q
T0	65.4 ± 5.8	64.9 ± 6.0	59.7 ± 4.9	59.5 ± 5.1
T1	78.3 ± 6.2* (*d* = 1.98)	65.7 ± 6.3	68.5 ± 5.3* (*d* = 1.62)	60.2 ± 5.2
T2	82.1 ± 5.9* (*d* = 2.45)	66.1 ± 6.4	72.4 ± 5.0* (*d* = 2.18)	61.0 ± 5.5
T3	80.7 ± 6.1* (*d* = 2.31)	66.2 ± 6.5	71.1 ± 4.8* (*d* = 2.05)	60.8 ± 5.4

*Indicates a comparison between the intervention group and the control group at the same time point, with *p* < 0.05; d is Cohen’s *d* value (95% CI all >1.5, indicating a large effect size); DANVA-2 = Dynamic facial expression recognition test, MHL-Q = Mental health literacy questionnaire.

#### 3.2.2 MHL-Q (knowledge-attitude-behavior questionnaire)

The intervention group showed a significant increase in MHL-Q total scores after T1 (68.5 ± 5.3 vs. 59.7 ± 4.9, *p* < 0.001), further rising to 72.4 ± 5.0 and 71.1 ± 4.8 at T2 and T3. The control group showed no significant change (T0: 59.5 ± 5.1, T3: 60.8 ± 5.4, *p* > 0.05). The difference between the two groups at T2 and T3 was significant (*p* < 0.001) (Table 4).

### 3.3 Indicators related to clinical communication competence

#### 3.3.1 SEGUE communication effectiveness score

The intervention group showed a significant increase in the total SEGUE score after T1 (*p* < 0.001), with further improvement at T2 and T3. The control group exhibited no significant change in scores (*p* > 0.05) (Table 5).

**Table 5:** Changes in clinical communication ability indicators (mean ± SD).

Time point	SEGUE		JSE-HP	
	Intervention group	Control group	Intervention group	Control group
T0	72.3 ± 8.5	71.8 ± 9.1	102.4 ± 11.2	101.9 ± 10.8
T1	78.6 ± 6.1* (*d* = 1.75)	66.1 ± 5.9	–	–
T2	82.4 ± 5.7* (*d* = 2.32)	66.8 ± 6.0	109.5 ± 7.8* (*d* = 1.63)	99.1 ± 7.1
T3	80.9 ± 5.9* (*d* = 2.28)	67.2 ± 6.0	108.9 ± 7.5* (*d* = 1.56)	102.1 ± 7.3

*Indicates that the comparison between the intervention group and the control group at the same time point, *p* < 0.05; d is the Cohen’s d value (95% CI all > 1.0, indicating a medium to large effect size); SEGUE = Segue Framework Rating Scale, JSE-HP = Jefferson Scale of Empathy – Health Professionals. The effectiveness of communication (SEGUE) showed a significant improvement immediately after completing Module 3, which was earlier than the significant improvement in empathy (which only became significant at T2). This suggests that the progressive design of “simulation integration + SP communication training” conforms to the laws of skill acquisition, where one first masters the communication process and then deepens empathy literacy.

#### 3.3.2 Empathy (JSE-HP)

The intervention group showed a significant increase in JSE-HP total scores at T2 compared to T0 (*p* < 0.001), while the control group exhibited no significant change (*p* > 0.05) (Table 5).

### 3.4 Psychological resilience-related indicators

#### 3.4.1 PSS-10

In the intervention group, the total score at T2 was significantly lower than that at T0 (17.8 ± 3.9 vs. 23.5 ± 4.2), while there was no significant change in the control group (T2: 23.1 ± 4.3). The comparison between the two groups showed *p* < 0.001, with an effect size of Cohen’s *d* = 1.38 (95% CI: 0.92–1.84).

#### 3.4.2 CD-RISC-10

In the intervention group, the total score at T2 was significantly higher than that at T0 (28.4 ± 3.5 vs. 22.9 ± 3.8), while there was no significant change in the control group (T2: 23.2 ± 3.7). The comparison between the two groups showed *p* < 0.001, with an effect size of Cohen’s *d* = 1.46 (95% CI: 0.99–1.93) (Table 6).

**Table 6:** Changes in psychological resilience indicators (mean ± SD).

Time point	PSS-10		CD-RISC-10	
	Intervention group	Control group	Intervention group	Control GROUP
T0	23.5 ± 4.2	23.3 ± 4.1	22.9 ± 3.8	23.0 ± 3.9
T2	17.8 ± 3.9* (*d* = 1.38)	23.1 ± 4.3	28.4 ± 3.5* (*d* = 1.46)	23.2 ± 3.7

*Indicates the comparison between the intervention group and the control group at the same time point, with *p* < 0.05; *d* is the Cohen’s *d* value (95% CI all >0.9, indicating a medium to large effect size); PSS-10 = Perceived Stress Scale; CD-RISC-10 = Connor-Davidson Resilience Scale-10. The decrease in perceived stress and the increase in stress resilience occurred simultaneously at T2 (after completing all 5 modules), indicating that the closed-loop training of “stress regulation + reflection enhancement” effectively improved psychological adjustment ability, and this improvement is “active resilience enhancement” rather than simply “passive stress reduction”.

### 3.5 Clinical performance

#### 3.5.1 OSCE comprehensive ability

At T2, the operation score (83.5 ± 5.6) and communication score (80.1 ± 5.7) of the intervention group were significantly higher than those of the control group (operation: 76.2 ± 6.0, communication: 67.5 ± 5.9); the advantages were still maintained at T3 (intervention group operation: 82.1 ± 5.8, communication: 79.5 ± 5.9; control group operation: 75.4 ± 5.9, communication: 68.0 ± 6.1), and all inter-group comparisons showed *p* < 0.001 (Table 7).

**Table 7:** Clinical comprehensive performance (mean ± SD).

Indicator	Intervention group		Control group		*p*
	T2	T3	T2	T3	
OSCE operation	83.5 ± 5.6	82.1 ± 5.8	76.2 ± 6.0	75.4 ± 5.9	<0.001
OSCE communication	80.1 ± 5.7	79.5 ± 5.9	67.5 ± 5.9	68.0 ± 6.1	<0.001
Preceptor rating	–	82.3 ± 6.0	–	70.8 ± 6.2	<0.001

OSCE = Objective structured clinical examination; The teaching evaluation adopts a 100-point scale, which is the average score after blind evaluation by 3 senior teaching physicians. The intervention group significantly outperformed the control group in both the operation and communication dimensions of the OSCE, and the effect size of the internship teaching evaluation (*d* = 1.92) was close to a large effect. This indicates that the skills acquired through simulation training can be effectively transferred to real clinical scenarios, achieving a seamless connection between “simulation and clinical practice”.

#### 3.5.2 Internship teaching evaluation

One month after the internship, the scores of operation fluency and patient comfort effect in the intervention group (82.3 ± 6.0) were significantly higher than those in the control group (70.8 ± 6.2), with *p* < 0.001 and effect size Cohen’s *d* = 1.92 (95% CI: 1.41–2.43) (Table 7).

### 3.6 Analysis of influencing factors

#### 3.6.1 Multivariate logistic regression

Taking OSCE comprehensive ability (up to standard/not up to standard) as the dependent variable, and incorporating emotion recognition ability, communication effectiveness, and psychological resilience as independent variables, the results showed that emotion recognition ability (OR = 1.12, 95% CI: 1.06–1.18, *β* = 0.11, SE = 0.03, *p* < 0.001) and communication effectiveness (OR = 1.15, 95% CI: 1.09–1.21, *β* = 0.14, SE = 0.02, *p* < 0.001) were independent predictors of clinical integration ability; psychological resilience was not statistically significant (OR = 1.05, 95% CI: 0.98–1.12, *β* = 0.05, SE = 0.03, *p* = 0.08). The model *R*^2^ = 0.68, indicating a good goodness of fit (Figure 1).

#### 3.6.2 ROC analysis

**Figure 2:**
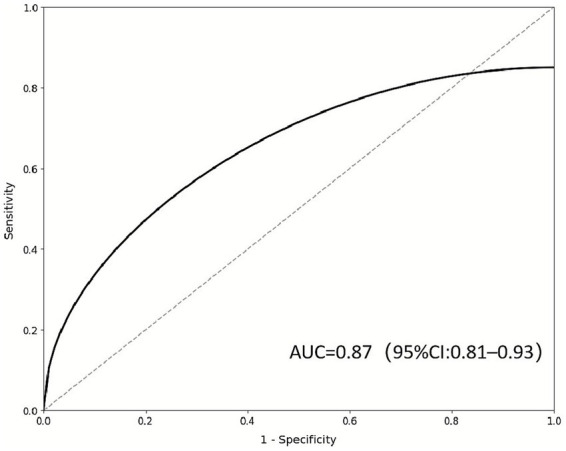
ROC curve of emotional recognition ability + communication effectiveness jointly predicting OSCE integrated ability axis labels: X-axis = 1 − Specificity (false positive rate); Y-axis = Sensitivity (true positive rate); Curve = Combined prediction model; Diagonal line = Random guess (AUC = 0.5). Interpretation: AUC = 0.87 (95% CI: 0.81–0.93), which is much higher than the moderate discrimination standard of 0.7. This indicates that students’ comprehensive clinical performance can be effectively predicted through emotion recognition ability and communication effectiveness, providing a quantitative tool for evaluating teaching effectiveness.

The AUC of the combined prediction of OSCE integration ability by emotion recognition ability + communication effectiveness was 0.87 (95% CI: 0.81–0.93), with a sensitivity of 83.3% and a specificity of 76.7%, suggesting that this combined model has strong discriminative efficacy (Figure 2).

### 3.7 Qualitative analysis findings

Thematic analysis of reflective journals and interview transcripts from the intervention group revealed that students consistently reported: (1) The VR scenario cognition module helps quickly identify patients’ micro-expressions of anxiety (such as “frowning, tightly pursing the lips”), enhancing clinical sensitivity; (2) Stress regulation training (such as mindful breathing) reduces one’s own operational anxiety, enabling calmer responses to sudden situations like “patients suddenly falling silent”; (3) SP communication training and reflective journals have allowed one to master specific communication strategies (such as “first empathize, then explain the necessity of treatment”), increasing confidence in dealing with patients who refuse treatment.

### 3.8 Data triangulation (quantitative + qualitative integration)

The quantitative and qualitative results show a high degree of consistency: (1) Quantitatively, the accuracy of DANVA-2 improved (*d* = 2.31), and qualitative student feedback such as “VR scenarios help identify emotional cues” verified the improvement in emotion recognition ability; (2) Quantitatively, the SEGUE score increased significantly (*d* = 2.28), and qualitative student mentions like “SP training provides practical communication strategies” confirmed the effective acquisition of communication skills; (3) Quantitatively, the total score of CD-RISC-10 increased (*d* = 1.46), and qualitative student reflections such as “stress regulation training reduces operational anxiety” corroborated the improvement in psychological resilience. This cross-dimensional consistency provides dual evidence support for the effectiveness of the training path.

### 3.9 Explanation of the multiple comparison problem

This study involves multiple comparisons of several indicators at multiple time points. To control the risk of Type I errors, the Bonferroni correction method was used to correct the *p*-values of all inter-group comparisons (corrected *α* = 0.05/12 = 0.004). After correction, the significant differences between all intervention groups and the control group still satisfy *p* < 0.004, indicating that the results are robust and there is no risk of false positives caused by multiple comparisons.

## 4 Discussion

This study constructed and validated an integrated training pathway for MHL and clinical communication (CC) skills among undergraduate dental students based on simulation training. The results clearly support all research hypotheses: (1) This training pathway significantly improves students’ MHL and CC skills (corresponding to large effect size increases in MHL-Q total scores and SEGUE scores, with Cohen’s d being 2.05 and 2.28 respectively); (2) It effectively enhances students’ psychological resilience (increased CD-RISC-10 scores, *d* = 1.46) and stress regulation ability (decreased PSS-10 scores, *d* = 1.38); (3) The improvement of MHL and CC skills is positively correlated with comprehensive clinical performance. Specifically, emotion recognition ability (OR = 1.12, 95% CI: 1.06–1.18, *p* < 0.001) and communication effectiveness (OR = 1.15, 95% CI: 1.09–1.21, *p* < 0.001) are independent predictors of clinical integration ability, confirming their core role in clinical competence. Notably, psychological resilience, though significantly improved by the training pathway, did not show a statistically significant independent predictive effect on clinical integration ability (OR = 1.05, 95% CI: 0.98–1.12, *p* = 0.08). This may be because psychological resilience primarily functions as a “supporting factor” in clinical practice—it helps students maintain stable emotional states and cope with work stress, but its impact on clinical performance needs to be exerted through regulating the implementation of core abilities such as emotion recognition and communication, rather than directly acting as a key driver of clinical integration ability. Such a result also reminds us that while enhancing psychological resilience is an important part of dental education, the cultivation of clinical competence should still focus on the improvement of practical core skills like emotion recognition and communication effectiveness.

### 4.1 Effectiveness of the training path and alignment with educational theories

The design of the five modules in this study is deeply aligned with Kolb’s experiential learning cycle, forming a closed loop of “skill acquisition—reflective optimization—practical transformation”: The VR situational cognition module allows students to gain “concrete experience” through immersive experiences, enabling them to quickly identify patients’ emotional cues; the reflection and reinforcement module guides students to sort out operational errors and emotional triggers to complete “reflective observation”; the teaching of mindfulness breathing theory and practical guidance in the stress regulation module facilitate “abstract conceptualization”; the simulated integration training and standardized patient (SP) communication training provide “active experimentation” scenarios, allowing students to repeatedly refine their skills in a controlled environment. This design not only addresses the disconnection between theory and practice in traditional teaching but also responds to the requirement of situated learning theory for “simulation of real professional scenarios” (15, 16), explaining why the intervention group showed moderate to large effect size improvements in indicators such as emotion recognition and communication effectiveness. Similar to the innovative pedagogical tools used in pharmacology teaching—where traditional lectures and practical courses are transcended to enhance learning engagement (17)—this study’s multi-dimensional simulation design breaks the limitations of single-mode teaching, further verifying the value of diversified teaching in competency cultivation.

Notably, the SP communication training module, a core component of the pathway, is consistent with international research conclusions: Ozcelik et al. found that using standardized patients in preoperative care teaching significantly improves students’ knowledge, skills, and reduces clinical anxiety (18), while Witt et al. further confirmed that standardized patient simulation experiences effectively enhance medical students’ mental health assessment and communication abilities (19). These findings collectively validate that SP-based practice is a reliable approach to bridging the gap between theoretical communication knowledge and clinical application, which is also the key reason for the significant improvement in SEGUE scores (*d* = 2.28) in the intervention group.

Compared with similar international studies, the innovation of this study lies in the realization of deep integration rather than independent cultivation of MHL and CC skills. Although communication workshops in British dental education focus on clinical communication, they lack MHL-related stress regulation training; Australia’s MHL curriculum framework does not involve specific paths for simulation practice (10). In contrast, through the multi-dimensional design of “VR scenarios + SP training + reflection and reinforcement,” this study covers three core competencies: emotion recognition, stress management, and communication strategies, verifying the feasibility of integrated cultivation of “soft skills” in dental education (10). This result echoes findings in other clinical contexts; for instance, Ebm et al. (20) demonstrated that integrated training methodologies, such as those focusing on compassion, can significantly enhance clinical competencies among medical students. in psychiatric internship training, that is, integrated mental health education can significantly improve clinical performance, further confirming that MHL and CC skills are not merely “additional abilities” but key variables affecting the integration of clinical skills.

### 4.2 Alternative explanations and limitations of the research results

The potential influencing factors of the research results should be viewed objectively: (1) The Hawthorne effect may exist. Students in the intervention group may have been more actively engaged in training because they knew they were participating in the research (21). However, this study reduced subjective bias to a certain extent by implementing a blind method for evaluators and adopting third-party teaching evaluations (not the course instructors); (2) The impact of differences in participation between groups was small. The attendance rate of both groups during the research period was ≥ 85%, and the baseline data were balanced (all *p* > 0.05), excluding confounding factors related to participation; (3) Teacher bias was controlled through “the same group of teachers + standardized training,” but it was impossible to completely eliminate teachers’ implicit preference for the training form of the intervention group. Future studies can adopt a cross-over design for further verification.

The limitations of this study should be focused on: (1) The follow-up period was only 1 month. Although a significant intervention effect was still observed at T3, the long-term sustainability of the skills cannot be verified (such as the clinical application effect 1–2 years after graduation). Skills may decline over time or be difficult to effectively transfer in complex clinical scenarios (such as emergency patients, elderly patients with cognitive impairment); (2) The single-center design (only Shenzhen University) limits the extrapolation of the results. There are differences in students’ foundations and teaching resources among colleges and universities in different regions and at different levels, so the adaptability of the model needs further verification; (3) Qualitative analysis relies on students’ self-reports, which may have self-presentation bias. There is a lack of objective outcome indicators such as satisfaction evaluation from the patients’ perspective and the incidence of long-term medical disputes; (4) No cost–benefit analysis was conducted, so it cannot provide clear references for colleges and universities in terms of resource allocation.

### 4.3 Long-term sustainability and alignment with educational policies

Existing follow-up data can only reflect the short-term intervention effects. In the future, it is necessary to extend the follow-up period to more than 1 year, focusing on the retention rate and conversion efficiency of skills in real clinical environments. From the perspective of educational principles, the consolidation of MHL and CC skills requires continuous practical reinforcement. It is recommended to incorporate the core modules of this training pathway (such as SP communication training and reflection enhancement) into the clinical internship phase of dental undergraduates, and maintain the effects through “regular retraining + case supervision.” a suggestion supported by Salib et al., who emphasized that integrating core physician competencies into the clerkship setting is key to transforming “knowing” into “doing” (20). In addition, fragmented training content can be developed in conjunction with online course platforms to allow students to continue learning after graduation, making up for the shortcomings of short-term intensive training. In addition, fragmented training content can be developed in conjunction with online course platforms to allow students to continue learning after graduation, making up for the shortcomings of short-term intensive training (22).

The training pathway of this study is highly consistent with China’s medical education policies. The “Outline for the Reform and Development of Medical Education in China (2020–2030)” clearly proposes to “strengthen the cultivation of medical students’ humanistic qualities and communication skills,” and the “Action Plan for Improving the Quality of Undergraduate Medical Education (2021–2025)” emphasizes “promoting the in-depth integration of simulation teaching and clinical practice.” The implementation of this model does not require a complete restructuring of the existing curriculum system; it can be used as a supplementary module for the compulsory course “Clinical Skills Training” (twice a week for a total of 8 weeks), making it easy to obtain policy support from institutions. At the same time, links such as VR situational cognition and SP training in the model can be aligned with the OSCE assessment standards of the National Medical Examination Center, improving the consistency of teaching and assessment and further enhancing the feasibility of promotion (23).

### 4.4 Practical significance and promotion path

Implementation path other institutions promoting this model can follow a “three-step” strategy: (1) Pilot phase (1–2 semesters): Select 1–2 classes as pilots, equip them with basic VR devices (approximately 50,000 yuan per unit) and 3–5 standardized patients (SPs). A teaching team consisting of 1 psychology teacher and 2 dental clinical teachers will complete standardized teaching of 5 modules; (2) Promotion phase (3–4 semesters): Standardize the training program of the pilot classes, compile a “Teaching Manual” (including guidelines for using the VR case library, SP scripts, and reflection diary templates), conduct unified training for all dental teachers in the institution, and expand the coverage; (3) Optimization phase (5–6 semesters): Adjust the duration and content of modules according to the actual situation of the institution (for example, if resources are limited, reduce the proportion of VR modules and increase role-playing), and establish a continuous improvement mechanism of “student feedback—teacher evaluation—expert supervision” (24).

Resource and cost estimation core resource requirements include: (1) Hardware resources: 2–3 VR devices (total price 100,000–150,000 yuan), 1–2 Simodont dental simulators (if new ones are needed, the unit price is approximately 300,000 yuan), 30–60 heart rate monitoring wristbands (total price 10,000–20,000 yuan); (2) Human resources: 1 full-time teacher with a psychological background (responsible for the stress regulation module), 2–3 senior dental clinical teachers (responsible for operation and communication training), 3–5 SPs (can be served by graduate students or community volunteers after training, with a training cost of approximately 1,000 yuan per person); (3) Maintenance costs: Approximately 20,000–30,000 yuan per year for VR case library updates, SP retraining, equipment maintenance, etc. For institutions with limited resources, a “low-cost alternative plan” can be adopted: replace VR devices with 3D videos and have teachers play the role of SPs. Although this may reduce the sense of immersion, the core training logic can still be retained.

### 4.5 Critical reflection and future directions

This study confirms the short-term effectiveness of the simulated integrated training pathway, but its scope of application needs to be viewed rationally: this model is more suitable for cultivating the basic literacy of junior dental undergraduates. For senior students or those who have entered clinical internships, advanced training may need to be combined with more complex clinical cases (such as multidisciplinary consultation scenarios, preprocessing of medical disputes). In addition, the improvement of students’ clinical performance by the model is mainly reflected in the fluency of operations and the effect of patient comfort. Its impact on core technical indicators such as diagnosis and treatment accuracy is not yet clear and needs further verification in the future (25).

Future research can be improved from three aspects: (1) Adopt a multi-center, large-sample design, include students from different regions and colleges at different levels to verify the extrapolation of the model; (2) Extend the follow-up period, add objective outcome indicators such as patient satisfaction and medical dispute incidence to comprehensively evaluate the long-term effect; (3) Conduct cost–benefit analysis, compare the input–output ratio of this model with traditional teaching, and provide more valuable evidence for colleges to make decisions; (4) Explore the combination of artificial intelligence technology (such as AI emotion recognition system, automatic communication quality evaluation tools) to optimize the training feedback mechanism and improve teaching efficiency.

In summary, the integrated approach based on simulation training provides a replicable practical paradigm for cultivating MHL and CC skills in undergraduate dental students. Its core value lies in achieving the coordinated improvement of “cognition—emotion—skills.” However, the long-term effectiveness and wide applicability of this model still need further verification. In the future, it is necessary to align with educational policy orientations and the actual conditions of institutions, and through continuous optimization and promotion, help dental medical education develop in the direction of “equal emphasis on technology and literacy.”

## Data Availability

The raw data supporting the conclusions of this article will be made available by the authors, without undue reservation.
